# Diaphorin, a polyketide produced by a bacterial symbiont of the Asian citrus psyllid, kills various human cancer cells

**DOI:** 10.1371/journal.pone.0218190

**Published:** 2019-06-10

**Authors:** Atsushi Nakabachi, Keiko Okamura

**Affiliations:** 1 Electronics-Inspired Interdisciplinary Research Institute (EIIRIS), Toyohashi University of Technology, Toyohashi, Aichi, Japan; 2 Department of Environmental and Life Sciences, Toyohashi University of Technology, Toyohashi, Aichi, Japan; US Department of Agriculture, UNITED STATES

## Abstract

Diaphorin is a polyketide produced by *Candidatus* Profftella armatura (*Betaproteobacteria*), an organelle-like defensive symbiont harbored by a plant sap-sucking insect, Asian citrus psyllid *Diaphorina citri* (Hemiptera: Liviidae). Diaphorin belongs to the pederin family, a group of compounds that share much of their core structure with that of pederin, which is characterized by two dihydropyran rings bridged by an *N*-acyl aminal. Most members of this family have potent antitumor activity, making them promising anticancer drug candidates. The present study assessed the therapeutic potential of diaphorin for its antitumor activity against 39 human cancer cell lines including those from breast, brain, colon, lung, skin, ovary, kidney, stomach, and prostate. The results showed that diaphorin had inhibitory activity against all 39 cancer cell lines tested. The GI_50_, TGI, and LC_50_ values ranged from 0.28 μM– 2.4 μM, 1.6 μM –11 μM, and 7.5 μM–> 100 μM, respectively. These values are among the highest in the pederin family, indicating that the anticancer activity of diaphorin is milder than those of other pederin congeners. The inhibitory effects of diaphorin significantly differed among the distinct cancer types. The maximum difference was about 10-fold, which was similar to those of most other pederin congeners.

## Introduction

A variety of invertebrate animals harbor endosymbiotic microbes to deter natural enemies, which makes them among the richest sources of biologically active secondary metabolites [1–6]. Pederin family compounds, mostly isolated from marine sponges, are polyketides that share much of their core structure with that of pederin, which is characterized by two dihydropyran rings bridged by an *N*-acyl aminal [7–12]. Most compounds in this family, including pederin, mycalamides, onnamides, theopederins, and psymberin, exhibit potent antitumor activity, making them promising as anticancer drug candidates [7–10,13–20]. Mycalamide A not only inhibited replication of various cancer cells *in vitro* but also improved the survival of mice bearing ascitic lymphomas and a variety of other ascitic and solid tumors [15]. Moreover, this compound exhibited cancer preventive properties, inhibiting epidermal growth factor (EGF)-induced neoplastic transformation in murine cells [20]. Analysis using 60 human cancer cell lines revealed that psymberin, also known as irciniastatin A [21], has an remarkably selective activity toward solid tumor cells, making it one of the most promising lead drug candidates among a variety of other possible leads from different structural classes [22].

**Fig 1:**
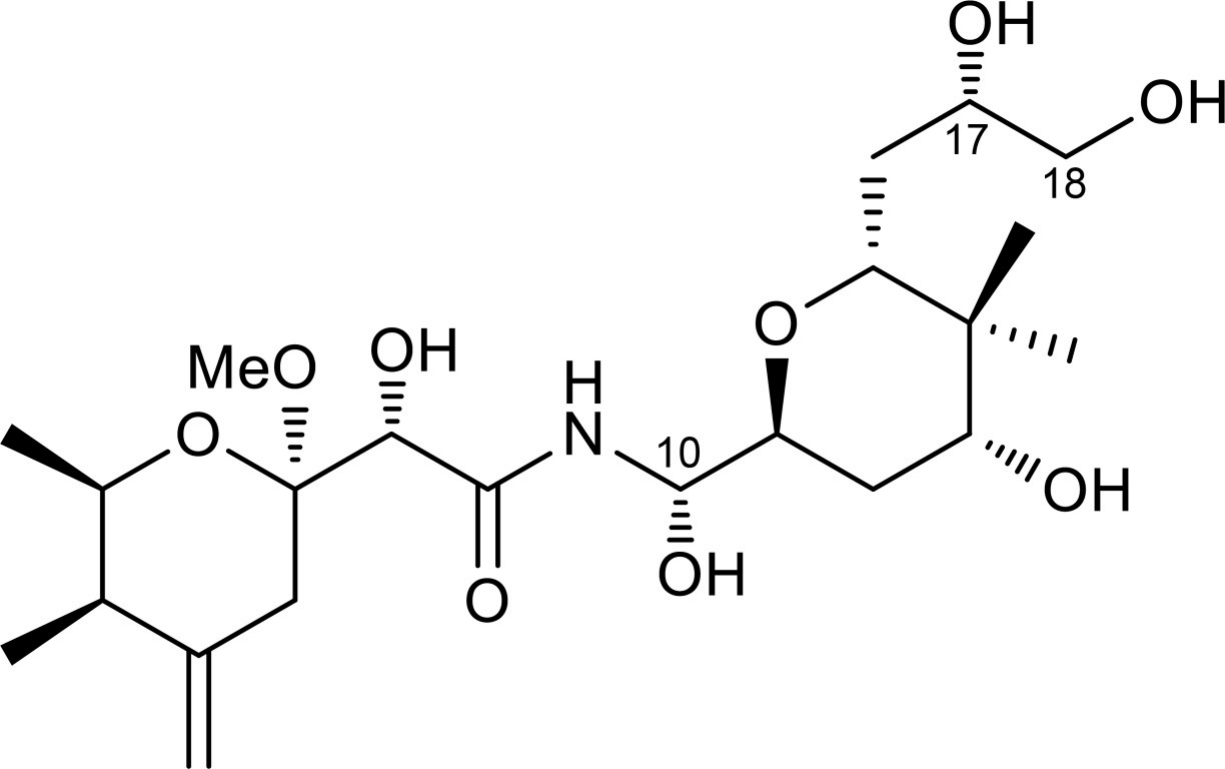
Chemical structure of diaphorin.

Although most pederin congeners are derived from marine invertebrates, pederin itself is found exclusively from rove beetles of the genera *Paederus* and *Paederidus* (Coleoptera: Staphylinidae) [7–10,23,24]. Since the discovery of pederin in the mid 20^th^ century [25], these beetles have long been a sole terrestrial source of pederin family members until diaphorin, a tri-*O*-desmethyl analog of pederin (Fig 1), was discovered from a plant sap-sucking insect, the Asian citrus psyllid *Diaphorina citri* (Hemiptera: Liviidae)[26]. *D*. *citri* is an important pest of citrus worldwide and harbors the vertically transmitted intracellular symbiont *Candidatus* Profftella armatura (*Betaproteobacteria*) [26–30]. *Profftella* is an unprecedented organelle-like defensive symbiont with a drastically reduced genome of 460 bp, which produces diaphorin using the polyketide synthase (PKS) biosynthetic gene clusters [26]. A previous study showed that diaphorin has significant inhibitory activity against the rat neuroblastoma B104 and human HeLa cells [26]. In the present study, in an effort to further evaluate the therapeutic potential of diaphorin, we assessed the antitumor activities of diaphorin against various human cancers using a panel of 39 cancer cell lines prepared by the Japanese Foundation for Cancer Research.

## Materials and methods

### Preparation of diaphorin

Diaphorin was extracted from *D*. *citri*, purified, and quantified as previously described [30]. Briefly, adult *D*. *citri* were treated twice with methanol, and the extracts were combined and concentrated *in vacuo*. The residue was resuspended in methanol and purified in a Shimadzu (Kyoto, Japan) LC10 high-performance liquid chromatography (HPLC) system with an Inertsil ODS-3 C18 reversed-phase preparative column [5 μm, 7.6 × 150 mm, GL Science (Tokyo, Japan)] heated to 35°C. The mobile phase was isocratic 20% acetonitrile in water, with a flow rate of 1.5 mL/min. Diaphorin was detected at a wavelength of 200 nm. The purified samples were combined and dried *in vacuo*. Diaphorin was re-dissolved in methanol and quantified in an HPLC system as described above, except the mobile phase was 15% acetonitrile in water, with a flow rate of 1.0 mL/min, and an Inertsil ODS-3 analytical column (5 μm, 4.0 × 250 mm, GL Science) was used.

### Cell lines and cell culture

The human cancer cell line panel prepared by the Japanese Foundation for Cancer Research (Tokyo, Japan) consisted of 39 cell lines as follows [31]: breast cancer, BSY-1, HBC-4, HBC-5, MCF-7, and MDA-MB-231; central nervous system (CNS) cancer, SF-268, SF-295, SF-539, SNB-75, SNB-78, and U251; colon cancer, HCC-2998, HCT-116, HCT-15, HT-29, and KM-12; lung cancer, A549, DMS114, DMS273, NCI-H226, NCI-H23, NCI-H460, and NCI-H522; melanoma, LOX-IMVI; ovarian cancer, OVCAR-3, OVCAR-4, OVCAR-5, OVCAR-8, and SK-OV-3; renal cancer, ACHN and RXF-631L; stomach cancer, MKN-A, MKN-B, MKN1, MKN45, MKN74, and St-4; prostate cancer, DU-145 and PC-3. Normal cell types were not included in the assay because they tend to respond to anticancer drugs with extreme phenotypes (e.g., fibroblasts being pan-resistant and renal epithelial cells being pan-sensitive), making them unsuitable as controls [32]. The cells were cultured in RPMI 1640 medium supplemented with 5% fetal bovine serum, 2 mM L-glutamine, penicillin (100 units/mL), and streptomycin (100 mg/mL) at 37°C in humidified air containing 5% CO_2_.

### Analysis of cell growth inhibition by diaphorin

Inhibition of the growth of human cancer cell lines was assessed using a standard sulforhodamine B (SRB) assay as described previously [33]. Briefly, cells in culture medium were inoculated into 96-well plates and incubated at 37°C for 24 h. Subsequently, 12 replicates of each cell line were fixed with trichloroacetic acid (TCA) to measure the cell population at the time of adding diaphorin (Tz: time zero). Serial dilutions (10^−8^, 10^−7^, 10^−6^, 10^−5^, or 10^−4^ M final concentration) of diaphorin were then added to each cell line. After 48 h of additional incubation at 37°C, the plates were fixed with TCA, stained with SRB, and read with an automated microplate reader at a wavelength of 515 nm. Percentage growth inhibition was calculated as:

[(Ti-Tz)/(C-Tz)] × 100, when Ti≥Tz

[(Ti-Tz)/Tz] × 100, when Ti<Tz

(Tz: the SRB absorbance at time zero, C: the SRB absorbance of the control cells receiving no diaphorin, Ti: the SRB absorbance of the test cells treated with diaphorin at the five concentration levels). The concentration of diaphorin resulting in a 50% reduction in the cell number increase as compared with the control (GI_50_: 50% growth inhibition) was calculated from [(Ti-Tz)/(C-Tz)] × 100 = 50. The concentration of diaphorin resulting in total growth inhibition (TGI) was calculated from Ti = Tz. The concentration of diaphorin resulting in a 50% reduction of the number of cells at the end of the treatment as compared with that at the beginning (LC_50_: 50% lethal concentration) was calculated from [(Ti-Tz)/Tz] × 100 = -50.

### Statistical analysis

Data were analyzed using R statistical computing software (version 3.4.2) [34].

## Results and discussion

### Growth inhibitory activity of diaphorin against 39 human cancer cell lines

**Fig 2:**
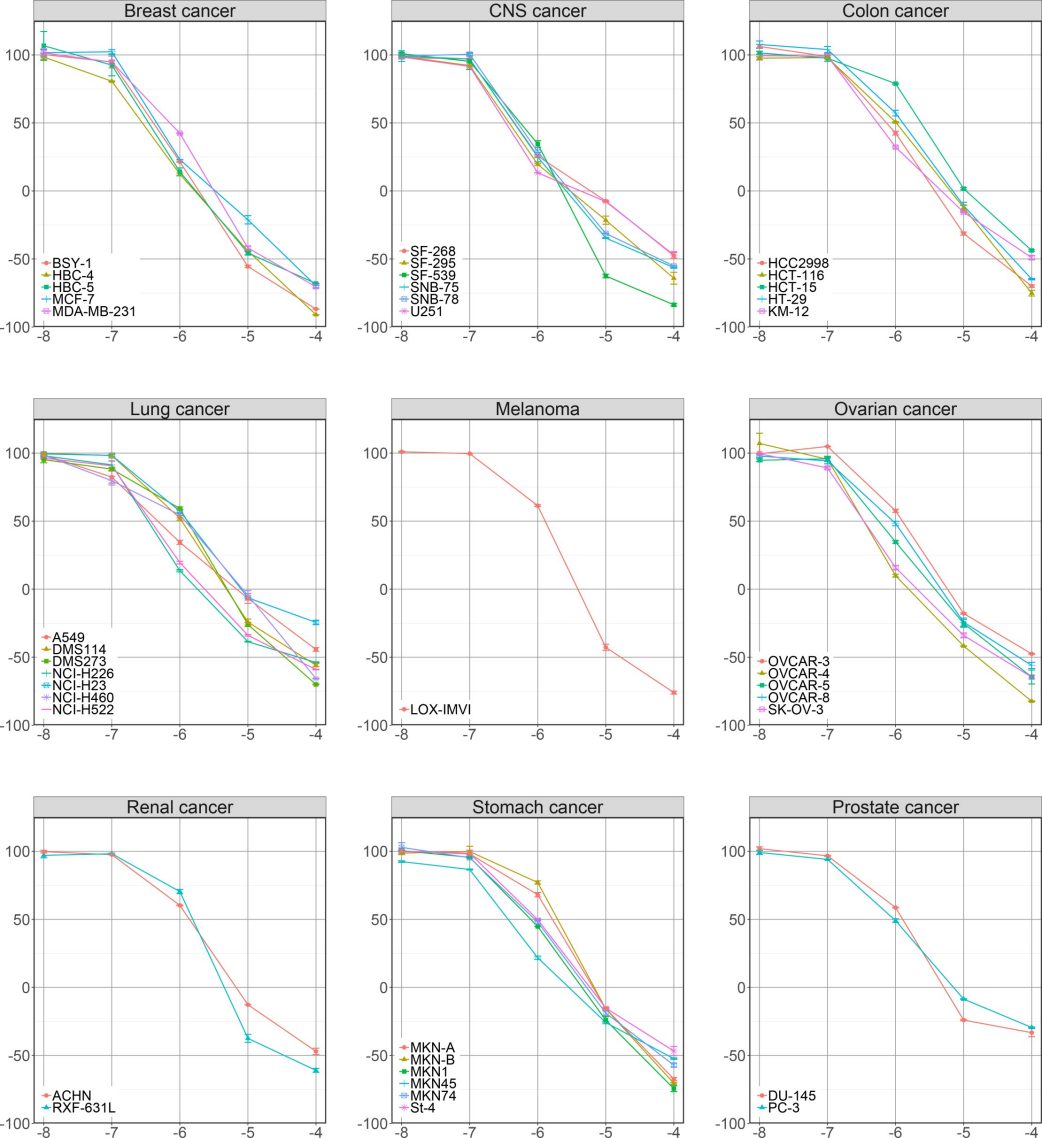
Dose response curves of the 39 human cancer cell lines treated with diaphorin.

A total of 39 human tumor cell lines were treated with diaphorin at final concentrations of 10^−8^, 10^−7^, 10^−6^, 10^−5^, and 10^−4^ M (S1 Table). The dose-response curves (Fig 2) showed that diaphorin had significant dose-dependent patterns of inhibitory activity against all 39 cancer cell lines tested (Tukey-Kramer test, *p* < 0.05). The concentrations of diaphorin required to exert the growth inhibitory activity were at the micromolar level, which is much higher than those reported for other members of the pederin family including pederin, mycalamides, onnamides, theopederins, psymberin, and their synthetic analogs that exhibit antitumor activity at nanomolar or even subnanomolar concentrations [9,10,13–20]. The anticancer activities of pederin congeners are primarily attributed to their ability to bind to ribosomes and inhibit protein synthesis [9,18,19,35,36]. Moreover, the C10 methoxy group of pederin and its analogs is postulated to be important for ribosome binding through its hydrogen bonding and its effects on conformation [9]. Diaphorin is a tri-*O*-desmethyl analog of pederin (Fig 1), where the methoxy groups at the C10, C17, and C18 of pederin are replaced by hydroxyl groups in diaphorin [26,37]. This alteration increases the hydrophilicity of the compound while enabling the occurrence of putative conformational changes and hydrogen bonding required for ribosome binding. Analyses of structure–activity relationships in this family support the idea that greater affinity toward ribosomes is associated with lower hydrophilicity [10], which most likely accounts for the notably milder activity of diaphorin compared with other pederin congeners.

The x-axis represents log10 molar concentrations of diaphorin added to the culture medium. The y-axis represents the percentage growth of the cells during the diaphorin treatment. The growth percentage value of 100 represents the growth of the control cells receiving no diaphorin, the value of 0 represents no net growth throughout the treatment period, and the value of -100 indicates that all cells are killed at the end of the experiment. Error bars represent standard errors.

**Fig 3:**
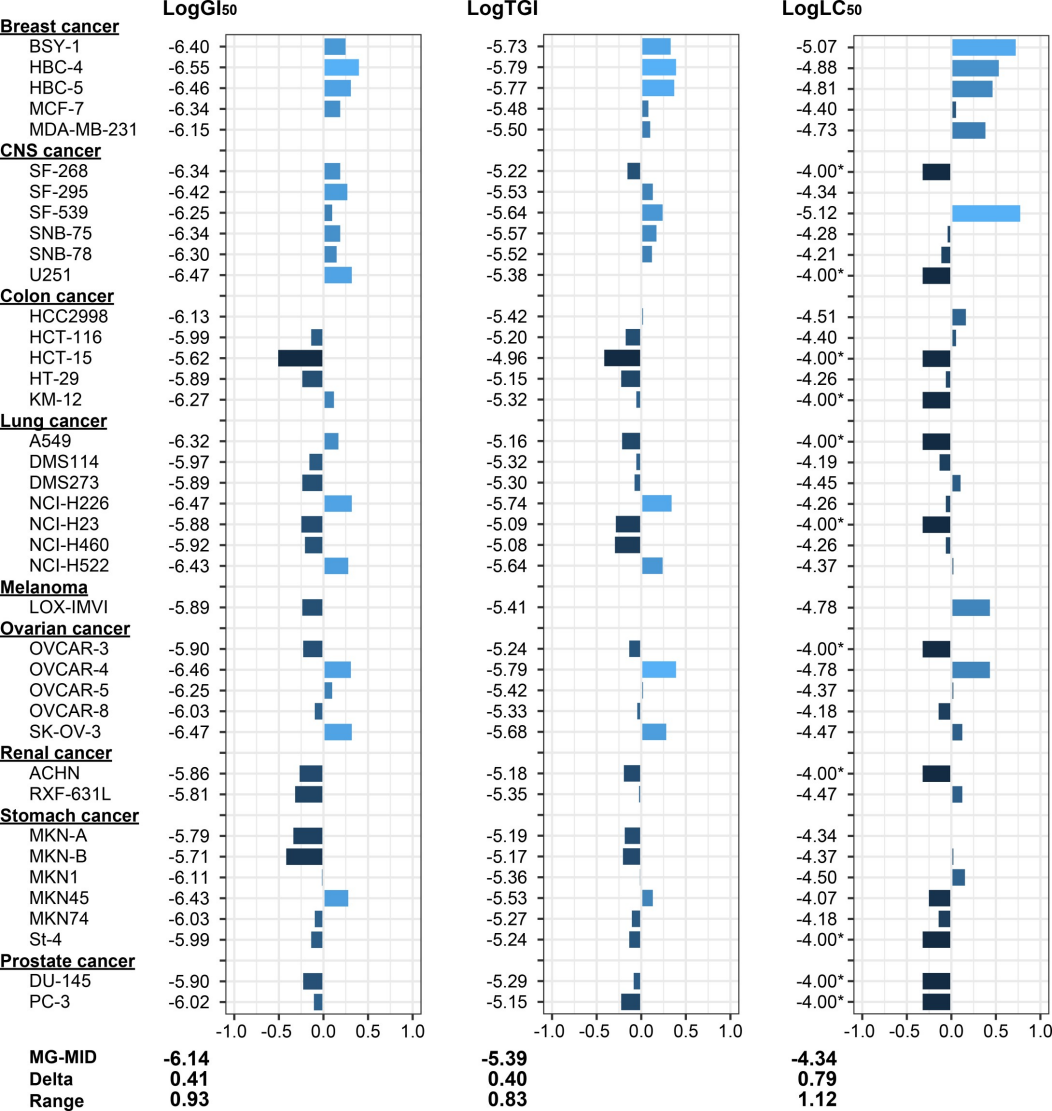
Mean graphs showing the relative sensitivity of each cell line against diaphorin. The logarithms of GI_50,_ TGI, and LC_50_ are indicated for each cell line. The x-axis represents the difference between the mean of the parameters for all 39 cell lines and the parameters for each cell line. Namely, columns extending to the right of center (mean) indicate that the corresponding cell lines are more sensitive to diaphorin than the average of the 39 cell lines, and columns extending to the left indicate that the cell lines are more resistant to diaphorin than the average. MG-MID: the mean of log GI_50,_ TGI, or LC_50_ for the 39 cell lines. Delta: the difference between the MG-MID and the log GI_50,_ TGI, or LC_50_ of the most sensitive cell line. Range: the difference between the log GI_50,_ TGI, or LC_50_ of the most resistant cell line and those of the most sensitive one. Asterisks (*) indicate that the log LC_50_ values were greater than -4.00 (10^−4^ M), the maximum concentration of diaphorin tested, at which the treatment did not result in 50% reduction of the cells at the end of the treatment as compared with those at the beginning.

The GI_50_, TGI, and LC_50_ values are summarized in Table 1. The GI_50_ values varied 10-fold, ranging from 0.28 μM for the HBC-4 breast cancer cells to 2.4 μM for the HCT-15 colon cancer cells. The TGI values ranged from 1.6 μM for the HBC-4 breast cancer cells and the OVCAR-4 ovarian cancer cells to 11 μM for the HCT-15 colon cancer cells. The LC_50_ values varied more than 10-fold; the most sensitive cell line was the SF-539 CNS cancer cells with an LC_50_ value of 7.5 μM followed by the BSY-1 breast cancer cells with an LC_50_ value of 8.4 μM. The least potent inhibitory activities with LC_50_ values greater than 100 μM were observed for the SF-268 and U251 CNS cancer cells, the HCT-15 and KM-12 colon cancer cells, the A549 and NCI-H23 lung cancer cells, the OVCAR-3 ovarian cancer cells, the ACHN renal cancer cells, the St-4 stomach cancer cells, and the DU-145 and PC-3 prostate cancer cells. In these cell lines, treatment with 100 μM diaphorin did not result in a 50% reduction of the cells at the end of the treatment as compared with that at the beginning. In terms of the three parameters, GI_50_, TGI, and LC_50_, the HCT-15 colon cancer cells were the least sensitive of the 39 cell lines. This is reasonable because HCT-15 is a multidrug-resistant cell line, which expresses the multidrug-resistance related genes MDR1 and MRP that are involved in the efflux of cytotoxic substances including therapeutic drugs [38]. The mean graphs of log GI_50,_ TGI, and LC_50_ visualize the relative sensitivity of each cell line against diaphorin (Fig 3).

**Table 1:** Indices showing the inhibitory activity of diaphorin against the 39 human cancer cell lines.

Cell line	GI_50_ (μM)	TGI (μM)	LC_50_ (μM)
Breast cancer			
BSY-1	0.40	1.9	8.4
HBC-4	0.28	1.6	13
HBC-5	0.35	1.7	16
MCF-7	0.46	3.3	40
MDA-MB-231	0.71	3.2	19
CNS cancer			
SF-268	0.46	6.0	>100
SF-295	0.38	2.9	46
SF-539	0.56	2.3	7.5
SNB-75	0.45	2.7	52
SNB-78	0.51	3.0	61
U251	0.34	4.2	>100
Colon cancer			
HCC2998	0.74	3.8	31
HCT-116	1.0	6.3	40
HCT-15	2.4	11	>100
HT-29	1.3	7.1	54
KM-12	0.54	4.7	>100
Lung cancer			
A549	0.47	7.0	>100
DMS114	1.1	4.8	65
DMS273	1.3	5.0	35
NCI-H226	0.34	1.8	55
NCI-H23	1.3	8.1	>100
NCI-H460	1.2	8.3	55
NCI-H522	0.37	2.3	43
Melanoma			
LOX-IMVI	1.3	3.9	17
Ovarian cancer			
OVCAR-3	1.3	5.8	>100
OVCAR-4	0.35	1.6	16
OVCAR-5	0.56	3.8	43
OVCAR-8	0.93	4.7	67
SK-OV-3	0.34	2.1	34
Renal cancer			
ACHN	1.4	6.6	>100
RXF-631L	1.5	4.5	34
Stomach cancer			
MKN-A	1.6	6.4	45
MKN-B	2.0	6.8	43
MKN1	0.78	4.4	32
MKN45	0.37	2.9	84
MKN74	0.93	5.3	67
St-4	1.0	5.8	>100
Prostate cancer			
DU-145	1.3	5.1	>100
PC-3	0.96	7.1	>100

The sensitivity toward diaphorin significantly differed among the distinct cancer types (Friedman test: Friedman chi-squared = 9.4821, df = 1, *p* = 0.002075). The maximum difference was about 10-fold, which is similar to those observed for most other pederin congeners. Psymberin is the only exception to this within the pederin family thus far, showing more than a 10,000-fold difference in its inhibitory activity toward distinct cancer types. Indeed, several melanoma, breast, and colon cancer cell lines were highly sensitive (LC_50_ < 2.5 nM) to psymberin, whereas six leukemia cell lines were invariably insensitive (LC_50_ > 25 μM), indicating a >10^4^-fold difference in inhibitory activities [22]. Psymberin is the only pederin congener that lacks the A-ring oxane and the 1, 3-dioxane ring, which may account for the remarkably selective inhibitory profile of this compound [22].

The present study revealed that diaphorin inhibits a wide variety of human cancer cells. The selectivity of the inhibitory effect of diaphorin against distinct cancer types was only moderate, suggesting that this compound is less promising as a drug lead compared with psymberin. However, the notably milder inhibitory activity of diaphorin compared with other pederin analogs may make it worthwhile to explore its use for other potential pharmaceutical applications.

## Supporting information

S1 Table Absorbance of sulforhodamine B at a wavelength of 515 nm. (XLSX)
